# Data Platform for Animal Mortality Information System (DATASIMA): Monitoring Companion Animal’s Euthanasia Causes in City of João Pessoa, Brazil

**DOI:** 10.3390/vetsci12010028

**Published:** 2025-01-08

**Authors:** Eduardo S. S. Sousa, Maria E. S. Sousa, Moisés D. C. A. Pereira, Ricardo A. M. Negreiros, Lilian R. C. Eloy, Arthur W. L. Brasil, Inácio J. Clementino, Sérgio S. Azevedo, Ricardo B. Lucena

**Affiliations:** 1 Graduate Program in Animal Health and Science, Center for Rural Health and Technology, Universidade Federal de Campina Grande, Patos 58708-110, Paraiba, Brazil; esergiosousa@uol.com.br; 2 Centre for Medical Sciences, Universidade Federal da Paraiba, João Pessoa 58051-900, Paraiba, Brazilricardo.negreiros@academico.ufpb.br (R.A.M.N.); 3 Nova Esperança College of Medicine and Nursing, João Pessoa 58051-900, Paraiba, Brazil; madu.rsc@gmail.com; 4 Agricultural Sciences Center, Universidade Federal da Paraiba, Areia 58397-000, Paraiba, Brazil; lilian.eloy@academico.ufpb.br (L.R.C.E.); inacio.clementino@acedemico.ufpb.br (I.J.C.); 5 Health Sciences Center, Universidade Federal da Paraiba, João Pessoa 58051-900, Paraiba, Brazil; arthurwilians7@yahoo.com.br; 6 Center for Rural Health and Technology, Universidade Federal de Campina Grande, Avenida Universitária, Patos 58708-110, Paraiba, Brazil; sergio.santos@professor.ufcg.edu.br

**Keywords:** animal mortality, epidemiological monitoring, health information systems, zoonotic disease surveillance

## Abstract

The literature regarding causes of animal mortality varies greatly in how it evaluates and describes reasons for euthanasia, showing a clear need for tools to identify, standardize, and map diseases. This study describes the application of the Animal Mortality Information System Database (DATASIMA) to monitoring and georeferencing animal mortality. An observational study was conducted on the dogs and cats euthanized at the Municipal Center for Environmental and Zoonotic Surveillance in João Pessoa, Paraíba. The deaths were reported through DATASIMA, an online platform created and developed in Brazil, which is aimed at observing animal mortality through the Animal Death Declaration between April and September 2022. A total of 403 euthanized animals were recorded, including 204 dogs and 199 cats. The most common diseases leading to euthanasia were as follows: (i) Sporotrichosis (171 cats); (ii) Leishmaniasis (75 dogs); (iii) Distemper (35 dogs); (iv) Trauma from vehicle collisions (ten dogs and nine cats). The DATASIMA platform proved to be an effective system for keeping records and reporting causes of animal mortality. Through georeferencing, it was possible to map areas with higher frequencies of zoonotic outbreaks in the municipality studied, thus providing useful data to implement health promotion strategies.

## Simple Summary

A new animal mortality monitoring platform, named DATASIMA, was utilized to track and record cases of animal euthanasia in the municipality of João Pessoa, Brazil. This system was designed to help understand why animals, such as dogs and cats, die or are euthanized, providing accurate data on their causes of death. From April to September 2022, 403 animals were euthanized at a Zoonoses Control Center in the city, with the most common reasons being infections like sporotrichosis in cats and leishmaniasis in dogs. Other causes included diseases such as canine distemper and injuries from vehicle accidents, which affect both species. DATASIMA also helped map areas where these health issues were most prevalent, offering valuable information for local health authorities to develop targeted health strategies and better address zoonotic disease outbreaks in the community.

## 1. Introduction

The development of methods or systems that enable the proper investigation and monitoring of animal health is essential for the adoption of appropriate hygiene practices as well as the implementation of measures aimed at preventing diseases in both animals and humans [1,2,3]. Therefore, accurately interpreting the causes of death in animals is crucial for developing public health strategies [4]. Countries where animal mortality surveillance systems are present [5,6,7,8,9] often surveil infectious diseases in farm animals. However, there are significant discrepancies in health monitoring across species, especially for companion animals. Additionally, there is no standardized international system for veterinarians who work in clinics and hospitals for reporting the causes of death of their patients, which hampers the identification of critical intervention points and often halts decision-making in animal health.

Unlike veterinary medicine, human medicine has national and international integrated mortality reporting systems, which standardize data provision and analysis. This allows the provision of objective data to entities responsible for epidemiological surveillance, which then profile deaths and develop public health intervention strategies [10].

In veterinary medicine, however, the available literature presents and describes the causes of animal death in a heterogeneous manner, with few reports on the creation of notification systems, data storage, or attempts to standardize information, which is an important limitation identified by researchers [11,12,13]. Although these causes are described differently, they can be grouped and standardized for better monitoring and inspection by entities interested in promoting animal health and welfare [14,15].

Euthanasia is an established procedure in veterinary medicine [16,17], which, according to the World Health Organization for Animal Health (OIE), is defined as “the act of inducing death using a method that causes a rapid and irreversible loss of consciousness with minimum pain and distress to the animal”. It is frequently employed for the control of zoonotic diseases [18]. Therefore, more robust data on different patterns of animal mortality within a specific region and timeframe may support decision-making regarding animal euthanasia [17]. The use of a surveillance system such as the Animal Mortality Information System Database (DATASIMA) provides a versatile tool for analyzing the causes of euthanasia and assessing the influence of epidemiological factors on animal death, enabling the monitoring of potential disease causes [19]. Through these notifications and data on case numbers and reasons for euthanasia, it will be possible to obtain epidemiological profiles and implement strong health surveillance measures. In Brazil, for example, one reason that justifies and allows euthanasia to be performed is the risk posed by severe zoonotic infections to public health [20].

In this context, the present study aims to describe the application of the DATASIMA platform in monitoring and georeferencing animal mortality related to euthanasia. A cross-sectional study was conducted to evaluate the epidemiological profile associated with these deaths, as well as the spatial mapping of their distribution in urban areas. Through this analysis, the study will contribute to outlining efficient public health strategies and responding to zoonotic outbreaks that may occur in each region while considering the realities and challenges encountered.

## 2. Materials and Methods

### 2.1. Study Design

This is a cross-sectional epidemiological observational study conducted at the Municipal Center for Environmental Surveillance and Zoonoses of João Pessoa (CMVAZ), Paraiba, Brazil. This is a public, free, and accessible service maintained by the Municipality of João Pessoa. The CMVAZ is responsible for monitoring zoonotic diseases in the municipality; however, pet owners also seek this service for clinical care of animals for any condition, not limited to zoonotic diseases. The study included the deaths of dogs and cats reported between April and September 2022. The study was developed and conducted using the guidelines suggested by the Strengthening the Reporting of Observational Studies in Epidemiology (STROBE) statement [21,22].

### 2.2. Animal Mortality Information System Data Platform (DATASIMA) and Data Collection

The data were extracted from the DATASIMA system software platform written in Portuguese, which were created and developed in Brazil for reporting animal deaths, and contributed to the Brazilian Unified Health System (Sistema Único de Saúde—SUS) [19]. DATASIMA is a new tool currently in the testing phase and has not yet been implemented as a mandatory requirement for animal health services. Adoption of this platform has been voluntary among veterinarians. For this research, data from all animals treated by the veterinarians at CMVAZ were utilized. This platform is intended for the notification and monitoring of animal mortality, whether due to spontaneous death or euthanasia. However, for this study, only cases related to euthanasia were compiled.

The information in DATASIMA is provided exclusively by veterinarians through a notification document called the “Animal Death Declaration” (“Declaração de Óbito Animmal”—DOA). The DOA describes the conditions that resulted in the animal’s death and categorizes the causes using an adapted version of the International Statistical Classification of Diseases and Related Health Problems, 10th Revision (ICD-10) [19,23]. The ICD was chosen for its comprehensive nature and established diagnostic criteria, which helped reduce the number of non-specific or intermediate causes of death, often referred to as “garbage codes” [24], and allowed more accurate and systematic assessment of animal mortality causes.

The platform form requires filling out 48 fields distributed across six sections: (i) Animal Guardian Identification; (ii) Animal Identification; (iii) Incident Identification; (iv) Likely Circumstances of Death; (v) Conditions and Causes of Death; (vi) Conclusion. To respect the privacy of guardians and the animals treated, the system allows complete access to the data only by local health authorities.

The data for each animal were compiled according to species (dog or cat), sex (female or male), and age. Regarding age, the animals were classified as newborns, kittens (for cats), puppies (for dogs), adults, or seniors, following an adaptation of the classification proposed by Fighera et al. and Togni et al. [25,26]. Additionally, based on their living conditions, animals were categorized as follows: domiciled animals with owners were classified as “household”. Animals living in community shelters with multiple animals were classified as “community”. Animals found moribund on the streets without a responsible owner were classified as “stray”. Finally, animals with no available information regarding their origin were classified as “unknown”.

A convenience sampling method was used, including all deaths resulting from euthanasia and reported during the six-month study period. Cases with incomplete or missing data were excluded. DATASIMA features a “Notification and Research Reports” function designed for auditing purposes and scientific contributions to veterinary health research. This function was used to retrieve the data utilized in the present study.

The causes of death were categorized into six pathological groups:

(i) Infectious and Parasitic Diseases: Diseases caused by bacteria, fungi, viruses, and parasites;
(ii) Neoplasms: Benign or malignant proliferations;
(iii) Natural Causes: Senility, without a diagnosis related to other causes;
(iv) External Causes: Trauma or intoxication;
(v) Pathophysiological Disorders: Metabolic, degenerative, or circulatory disorders;
(vi) Indeterminate: Undefined cause.

### 2.3. Ethical Aspects

This study was approved by the Ethics and Research Committee of the Center for Medical Sciences at the Universidade Federal da Paraiba, under approval code CAAE 44552621.0.0000.8069, and is backed by Resolution 196/96 of the National Health Council. The guardians of the animals included in the research, as well as the veterinarians who contributed to data collection, signed the Informed Consent Form.

### 2.4. Statistical Analysis

The object of analysis in this study included the following information about the animals: sex, estimated age, guardian’s address, cause, and nature of death. Data were extracted from DATASIMA in .CSV format and subsequently stored in spreadsheets. Qualitative data were presented using absolute and relative frequencies. Georeferencing was performed using the Kernel map technique, generated from the DATASIMA system, with color variations indicating case density in each region, utilizing a radius of 1.94 km^2^.

## 3. Results

### 3.1. General Causes of Death

A total of 403 animals were included in the present study: 204 were dogs, and 199 were cats. The epidemiological characteristics of the dogs’ deaths are presented in Table 1, while those of the cats are shown in Table 2; they were categorized according to general conditions. The highest number of cases was attributed to infectious and parasitic diseases, followed by neoplasms, natural causes, external causes, physiopathological disorders, and undetermined causes. Table 3 and Table 4 detail each reason for euthanasia in dogs and cats, respectively, according to the ICD-10 classification.

### 3.2. Frequent Causes of Death Characteristics

The DATASIMA platform revealed that the most frequent cause of death linked to euthanasia was sporotrichosis, affecting only cats (171 cases). Of these cases, only three were community animals, 50 were stray cats, and 112 were household cats. Most of the affected cats were adults (148) and males (129), and the majority had a previous laboratory diagnosis for the disease (137).

Leishmaniasis, the second most frequent cause of death in the study sample, was observed only in dogs with owners (75 cases), indicating they were household pets. Most of the affected dogs were adults (46), females (36), and 70 had a previous laboratory diagnosis of the disease.

As for deaths classified as “Indeterminate Cause” (22 cases) in this study, it should be noted that while these cases lacked an identified etiological cause, the animals experienced significant suffering, prompting the recommendation for euthanasia.

### 3.3. Georeferencing of Causes of Death of Animals

**Figure 1:**
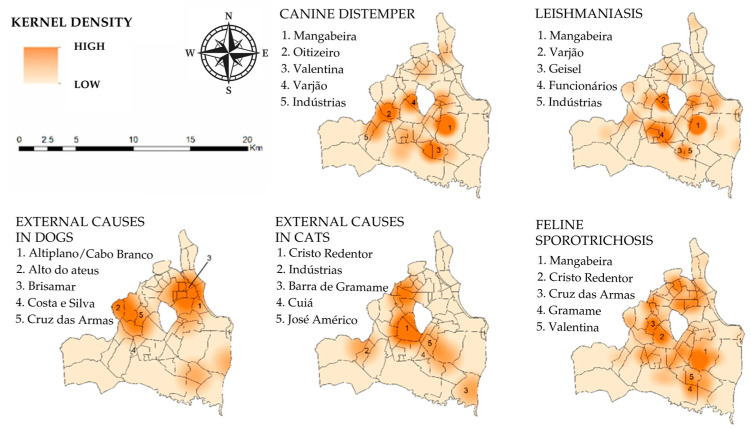
Georeferencing of the frequency of the main causes of euthanasia in dogs identified by the DATASIMA platform, according to the neighborhood/territory of the municipality of João Pessoa, Paraíba, Brazil. The top five neighborhoods for each condition/disease are listed below.

The causes of death, according to the places where these animals were kept when they became ill, were georeferenced. This allowed for the observation of the spatial distribution of diseases resulting in animal euthanasia, particularly those associated with zoonotic pathogens, across different neighborhoods in João Pessoa, Paraíba, Brazil. (Figure 1).

## 4. Discussion

The application of the DATASIMA platform effectively allowed a detailed analysis of the caseload related to animal deaths at the CMVAZ in João Pessoa, Brazil. Through this database, several characteristics of the death notifications were observed. The sample was properly characterized, highlighting important epidemiological aspects of the data analyzed. It is important to emphasize that it is viable to categorize, enumerate, and standardize information regarding the diagnoses leading to euthanasia through the ICD-10 classification. Significant zoonotic diseases are frequently diagnosed in companion animals in Brazil [27]. In this study, the georeferencing tool identified neighborhoods and epidemiologically sensitive areas where these diseases were present, highlighting regions that require targeted attention from municipal health surveillance institutions. The most common causes of death related to euthanasia in this study were sporotrichosis and leishmaniasis. The first is a significant disease in cats [28], while the latter is a frequent disease in dogs across different regions of Brazil [29,30,31]. It is important to note that the two most common causes are zoonotic diseases—highlighting the constant need for epidemiological surveillance through systems such as DATASIMA. The prevention of zoonotic diseases involves monitoring and evaluating the endemicity of a region, identifying transmission routes, and characterizing animal reservoirs of these diseases [3,32,33]. Brazil, which is considered a country with a health system capable of managing infectious diseases and zoonoses through the One Health approach [34], is an example of a nation that can benefit both regionally and nationally from the georeferencing tools available through DATASIMA, enabling connections between human and animal data, thereby contributing to the operation of the Brazilian Unified Health System—SUS.

In October 2021, Brazil enacted Law No. 14228, which prohibits the elimination of dogs and cats by zoonosis control agencies, public kennels, and similar official institutions, except in cases of severe or incurable contagious diseases that threaten human and animal health [35]. This law establishes important guidelines; however, certain practical gaps raise questions, and euthanasia often remains the only option when dealing with zoonotic diseases such as canine leishmaniasis or feline sporotrichosis, as demonstrated by the findings of the present study. According to the law, the determination that a disease is dangerous or incurable must be made by an official or private veterinarian with a valid professional license. In general, this conclusion should follow a thorough clinical evaluation, which is supported by complementary tests confirming the diagnosis and the severity of the condition.

It is important to note that the decision to perform euthanasia, although influenced by the pet owner, is not solely dependent on their will. The veterinarian must assess and justify the need for euthanasia. If the owner insists on euthanasia without legal justification, the veterinarian may refuse to perform the procedure. However, this is a complex ethical and practical issue, particularly because Brazil lacks a unified public health system for animals (a “SUS for animals”), unlike the healthcare system available for human diseases.

When owners lack financial resources to treat their animals, possible alternatives include assistance from nonprofit organizations or low-cost clinics offering free or affordable treatments. Nevertheless, these options fall far short of meeting the demand. Another possibility is finding a new family or organization capable of covering the treatment costs. Regardless, the legal gap creates practical dilemmas, as the law does not provide practical solutions for owners in vulnerable situations.

Sporotrichosis was the most frequently diagnosed disease in this study and, thus, the leading cause of euthanasia in the sample. Although this zoonotic disease is treatable, many cases may be refractory to treatment. Furthermore, a lack of information among pet owners about early treatment options often results in severe and widespread skin disease in affected cats. This mycosis, caused by dimorphic fungi of the genus *Sporothrix*, affects various mammalian species, with cats being the most affected. It frequently occurs in urban areas under epidemic conditions [36], with *Sporothrix brasiliensis* emerging as a prevalent species in Brazil [37]. Cats exhibit unique clinical signs that facilitate the multiplication of the pathogen, which promotes transmission. Brazil has the highest incidence of human sporotrichosis in Latin America, primarily due to hyperendemic feline-associated human sporotrichosis [38].

Based on the findings of the present study, sporotrichosis demands attention in urban areas where the disease appears more frequently. In our case series, most of the affected animals were male, adult, domesticated cats with guardians. This raises concerns since these cats live in close contact with humans, increasing the risk of zoonotic transmission [38].

The DATASIMA platform revealed that most leishmaniasis-related deaths occurred in domesticated dogs with owners. This close contact with the community increases the risk of zoonotic outbreaks and highlights the potential for rising human infection cases [29]. Georeferencing has identified areas requiring greater attention from health authorities for surveillance activities.

In Brazil, the domestic dog (*Canis familiaris*) is the primary reservoir of the etiological agent of Visceral Leishmaniasis (VL) in urban areas, with canine infection being more prevalent than human infection [39,40]. The disease in dogs spreads quickly when there are optimal transmission conditions, such as high density of insect vectors and susceptible animals. The seroprevalence of dogs with Visceral Leishmaniasis (VL) is significantly higher than the prevalence of symptomatic animals with cutaneous or mucocutaneous disease [39,41]. Asymptomatic dogs are also capable of transmitting the etiological agent of VL to insect vectors [42].

The release of SisLeish in 2013 by PAHO marked a significant step in the fight against leishmaniasis in the Americas [43]. This system aims to consolidate high-quality data, providing essential epidemiological information for health management. With the participation of 17 countries (except French Guiana), SisLeish enables comprehensive analysis of leishmaniasis data at various geographic levels, facilitating regular monitoring of cases [44].

A study published in 2017 using SisLeish indicates that the use of monitoring tools was crucial to prioritize areas, support decision-making processes, and guide disease surveillance and control efforts in the participating countries [44]. Therefore, it is relevant to compare the efficiency of the platform, which exclusively manages leishmaniasis data, with the potential of DATASIMA. DATASIMA does not provide detailed analysis and information regarding only a single disease but also comprehensively monitors all possible etiological causes related to animal mortality.

Other monitoring systems, both globally and locally, have been developed to track animal diseases. For example, the World Animal Health Information System (WAHIS), managed by the WOAH, focuses on identifying disease outbreaks in domestic and wild animals [45,46]. However, this system does not specifically address companion animals. Additionally, data collection from contributors requires periodic updates, making real-time monitoring impossible. Another limitation is that the accuracy of the data depends on the organization and commitment of governments, which can result in inaccurate reports [45].

An example of such a limitation can be seen in Brazil’s Zoosanitary Information System (SIZ), which monitors notifications of diseases and animal deaths. The national system in use is the E-Sisbravet, designed to monitor diseases listed in the Brazilian Ministry of Agriculture’s Normative Instruction No. 50/2013 [47]. This includes diseases that are not present in certain regions, diseases subjected to official surveillance programs, or those that require official intervention for control or eradication, as well as sporadic, exotic, and emerging diseases. However, this system mainly focuses on commercially important species and on the notifiable diseases list; these are only diseases from production animals [48].

Another system developed by Brazilian researchers is the ’Sistema Urubu’ (www.sistemaurubu.com.br, accessed on 10 December 2022), which specifically focuses on monitoring wildlife deaths on roads [8]. This has been an important data source that reduces sampling bias in the identification of species hit on highways. Before any data are made publicly available through the app, photos submitted by citizens are analyzed by taxonomy experts for species identification. Each record includes a photograph of the animal, along with geographic coordinates and the date it was taken [49]. However, the information is collected by citizens rather than professionals, who may not identify the actual cause of the animal’s death or detect zoonotic agents.

Such shortcomings highlight the importance of a tool like DATASIMA, which was designed for continuous monitoring of events related to animal death, offering georeferencing data in addition to standardized reports through the Animal Death Certificate (DOA). DATASIMA stands out from other animal mortality data collection instruments as it is filled and notified by veterinarians. Instruments like the ’End of Life Survey’, which allows the collection of data by pet owners on the death of domestic dogs, enable the record of scientifically relevant aspects of animal health care. However, they are limited due to the absence of information input from veterinary professionals [13].

There is a growing emphasis on animal mortality surveillance, using it as an indicator of good animal health and building attention toward human–animal–nature interactions [50]. DATASIMA’s innovative features, such as standardization, easy access, the ability to generate auditable reports, and real-time georeferencing, highlight the system’s potential.

When evaluating the free-roaming animals mapped by the study, it was found that death by road traffic accidents was the most prevalent cause. These findings align with the literature, which shows that, in most cases, free-roaming or abandoned animals are more susceptible to being hit by vehicles [51].

The study has some limitations, such as the low number of cases due to the short data collection period and the limited number of veterinarians who used the application during its initial implementation phase. Another limitation is that data collection is restricted to a single service point. DATASIMA is not an official government system; it was recently developed and is still in the testing phase. Thus, veterinarians are not required to report cases of euthanasia or death of companion animals.

Although zoonosis centers in Brazil serve as references for diagnosing and controlling diseases and provide services to all neighborhoods within a municipality, they do not cover cases handled by private veterinarians. This condition limits the dataset of the present study, reflecting only a local reality [52]. However, studies with similar limitations are common in the global literature. Therefore, initiatives that aim to encourage the reporting of animal diseases, regardless of species or the cause of the disease, are crucial for understanding disease prevalence.

Nevertheless, the local data presented in this study can serve as a starting point to help design additional studies with a larger population of animals, given that DATASIMA offers extended surveillance. In this way, an integrated, standardized, and scalable online system like DATASIMA has the potential for national and even international monitoring.

Further studies are needed to map other characteristics and detail risk factors for specific areas, as the death notifications studied here contain limited information. It is highly likely that additional data, such as the owner’s socioeconomic status, the animal’s habits, and health conditions, could shed light on possible hypotheses about the deaths and the affected populations.

Some disease notifications had to be adapted since they are specific to animals and do not have a designated code in the ICD-10 [53]. For canine distemper, a systemic viral condition that also causes immunosuppression [54], code B34.8—Other viral infections of unspecified location—was used. For feline immunodeficiency and feline leukemia, ICD code C95.0—Acute leukemia of unspecified cell type—was employed. This adjustment introduces a weakness in the system, as the pathogenesis of these conditions is unique, and a human code would not represent animal pathogens in some cases. Therefore, the standardization system should be improved so that such specific situations are recognized, reported, and given the required importance.

## 5. Conclusions

The application of the DATASIMA platform for analyzing animal mortality due to euthanasia proved to be effective through the features of the system. It functions as an efficient tool for recording, reporting, and studying the causes of animal death, enabling data standardization and fostering a universal dialogue among veterinarians.

In comparison to available research in the literature, DATASIMA holds significant potential for developing mortality surveillance within the national health system, such as Brazil’s Unified Health System (SUS), and may contribute to One Health discussions regarding the human–animal–environment interface.

The most frequent causes of death identified were sporotrichosis, leishmaniasis, canine distemper, and road traffic accidents. The spatial distribution of deaths due to these diseases, which is identified through georeferencing, highlighted the presence of more sensitive areas prone to zoonotic disease outbreaks. In João Pessoa, the same neighborhoods were recurrently affected; these data facilitate epidemiological surveillance strategies and contribute to various future research efforts.

## Figures and Tables

**Table 1:** Epidemiological categorization of dogs euthanized in 2021 at the Municipal Center for Environmental Surveillance and Zoonoses of João Pessoa, Brazil; mapped by DATASIMA.

Variables	Total N (%)	Infect. and Parasitic N (%)	Neopl. N (%)	Natural Causes N (%)	External Causes N (%)	Indeterm. N (%)	Physiopathol. Disord. N (%)
Age Range							
Newborn	10 (5)	7 (5.8)	0 (0)	0 (0)	0 (0)	1 (7.1)	2 (10)
Puppy	15 (7)	12 (10.0)	0 (0)	0 (0)	1 (8.3)	1 (7.1)	1 (5)
Adult	106 (52)	74 (61.7)	7 (26.9)	0 (0)	10 (83.3)	5 (35.7)	10 (50)
Senior	73 (36)	27 (22.5)	19 (73.1)	12 (100)	1 (8.3)	7 (50.0)	7 (35)
Living Conditions							
Household	170 (83)	106 (81.5)	25 (96.2)	11 (91.7)	2 (16.7)	9 (64.3)	17 (85)
Stray	32 (16)	13 (10.0)	1 (3.8)	1 (8.3)	9 (75.0)	5 (35.7)	3 (15)
Unknown	2 (1)	11 (8.5)	0 (0)	0 (0.0)	1 (8.3)	0 (0.0)	0 (0)
Sex							
Female	87 (43)	48 (40.0)	14 (53.8)	14 (33.3)	5 (42.0)	8 (57.1)	10 (50)
Male	110 (54)	66 (55.0)	12 (46.2)	12 (66.7)	6 (50.0)	6 (42.9)	10 (50)
Indeterminate	7 (3)	6 (5)	0 (0)	0 (0)	1 (8)	0 (0)	0 (0)

**Table 2:** Epidemiological categorization of cats euthanized in 2021 at the Municipal Center for Environmental Surveillance and Zoonoses of João Pessoa, Brazil; mapped by DATASIMA.

Variables	Total N (%)	Infect. and Parasitic N (%)	Neopl. N (%)	Natural Causes N (%)	External Causes N (%)	Indeterm. N (%)	Physiopathol. Disord. N (%)
Age Range							
Newborn	7 (3.5)	5 (2.9)	1 (20)	0 (0)	0 (0)	1 (50)	7 (3.5)
Kitten	22 (11.1)	18 (10.3)	0 (0)	1 (11)	2 (25)	1 (50	22 (11.1)
Adult	163 (81.9)	146 (83.4)	3 (60)	8 (89)	6 (75)	0 (0)	163 (81.9)
Senior	7 (3.5)	6 (3.4)	1 (20)	0 (0)	0 (0)	0 (0)	7 (3.5)
Living Conditions							
Household	130 (65,3)	114 (65.1)	5 (100)	3 (33)	6 (75)	2 (100)	130 (65.3)
Community	3 (1,5)	3 (1.7)	0 (0)	0 (0)	0 (0)	0 (0)	3 (1.5)
Stray	58 (29,1)	50 (28.6)	0 (0)	6 (67)	2 (25)	0 (0)	58 (29.1)
Unknown	8 (4)	8 (4.6)	0 (0)	0 (0)	0 (0)	0 (0)	8 (4)
Sex							
Female	50 (25.1)	41 (23.4)	1 (20)	2 (22.2)	5 (62.5)	1 (50)	50 (25.1)
Male	143 (71.9)	129 (73.7)	4 (80)	6 (66.7)	3 (37.5)	1 (50)	143 (71.9)
Indeterminate	6 (3)	5 (2.9)	0 (0)	1 (11.1)	0 (0)	0 (0)	6 (3)

**Table 3:** Causes of euthanasia of dogs attended to at the Municipal Center for Environmental Surveillance and Zoonoses of João Pessoa; mapped by DATASIMA and classified according to the adaptation of the International Classification of Diseases, 10th Revision (ICD-10).

ICD-10 Classification	Number of Cases	Frequency
Infectious and Parasitic Diseases	data	
A77.4—Ehrlichiosis	1	0.5%
A82.0—Rabies	1	0.5%
B34.8—Other viral infections of unspecified location (Distemper)	35	17.2%
B55.0—Visceral leishmaniasis	74	36.3%
B55.1—Cutaneous leishmaniasis	1	0.5%
B60.0—Babesiosis	1	0.5%
B87.0—Cutaneous myiasis	4	2.0%
N71.0—Acute inflammatory disease of the uterus	1	0.5%
U04.9—Severe acute respiratory syndrome (SARS), unspecified	3	1.5%
Neoplasia		
C20—Malignant neoplasm of rectum	1	0.5%
C22.0—Hepatocellular carcinoma	1	0.5%
C40.0—Malignant neoplasm of scapula and long bones of upper limbs	1	0.5%
C41.0—Malignant neoplasm of bones of skull and face	2	1.0%
C50.0—Malignant neoplasm of mammary gland	9	4.4%
C62.9—Malignant neoplasm of testis, unspecified	1	0.5%
C76.0—Malignant neoplasm of head, face, and neck	6	2.9%
C78.0—Secondary malignant neoplasm of lungs	2	1.0%
R22.0—Localized swelling, mass, or lump of the head	2	1.0%
C83.0—Diffuse non-Hodgkin lymphoma, small cells (diffuse)	1	0.5%
Natural Causes		
R54—Senility	12	5.9%
External Causes		
T62.8—Toxic effect of other harmful substances ingested as food	1	0.5%
X49.0—Accidental poisoning by/and exposure to other and unspecified chemicals—residence	1	0.5%
S32.0—Fracture of lumbar vertebra	1	0.5%
T07—Unspecified multiple injuries	2	1.0%
T11.0—Superficial injury of the upper limb, unspecified level	1	0.5%
V09.3—Pedestrian injured in unspecified road traffic accident	6	2.9%
Indeterminate Causes		
Ignored	1	0.5%
R99—Other ill-defined and unspecified causes of mortality	13	6.4%
Pathophysiological Disorders		
D46.4—Unspecified refractory anemia	1	0.5%
R63.2—Anorexia	2	1.0%
G40.0—Idiopathic epilepsy and epileptic syndromes defined by location (focal) (partial) seizures of focal onset	1	0.5%
I50.0—Congestive heart failure	1	0.5%
K76.0—Fatty liver degeneration, not classified elsewhere	2	1.0%
K92.2—Gastrointestinal hemorrhage, unspecified	1	0.5%
L98.0—Sterile pyogranuloma	1	0.5%
M06.9—Rheumatoid arthritis, unspecified	1	0.5%
N18.0—End-stage renal disease	2	1.0%
R64—Cachexia	8	3.9%

**Table 4:** Causes of euthanasia of cats attended to at the Municipal Center for Environmental Surveillance and Zoonoses of João Pessoa, Brazil; mapped by DATASIMA and classified according to the adaptation of the International Classification of Diseases, 10th Revision (ICD-10).

ICD-10 Classification	Number of Cases	Frequency
Infectious and Parasitic Diseases		
A41.0—Septicemia due to Staphylococcus aureus	1	0.5%
U04.9—Severe acute respiratory syndrome (SARS), unspecified	1	0.5%
B42.9—Sporotrichosis	171	85.9%
N71.0—Acute inflammatory disease of the uterus	1	0.5%
B82.9—Unspecified intestinal parasitosis	1	0.5%
Neoplasia		
C50.0—Malignant neoplasm of nipple and areola	1	0.5%
C95.0—Acute leukemia of unspecified cell type	4	2.0%
External Causes		
V09.3—Pedestrian injured in unspecified road traffic accident	9	4.5%
Indeterminate Causes		
R99—Other ill-defined and unspecified causes of mortality	8	4.0%
Pathophysiological Disorders		
K62.3—Rectal prolapse	1	0.5%
R56.0—Febrile convulsions	1	0.5%

## Data Availability

The raw data supporting the conclusions of this article will be made available by the authors on request.
